# DNA Hydroxymethylation Levels Are Altered in Blood Cells From Down Syndrome Persons Enrolled in the MARK-AGE Project

**DOI:** 10.1093/gerona/glx198

**Published:** 2017-10-21

**Authors:** Fabio Ciccarone, Elisabetta Valentini, Marco Malavolta, Michele Zampieri, Maria Giulia Bacalini, Roberta Calabrese, Tiziana Guastafierro, Anna Reale, Claudio Franceschi, Miriam Capri, Nicolle Breusing, Tilman Grune, María Moreno‐Villanueva, Alexander Bürkle, Paola Caiafa

**Affiliations:** 1 Department of Biology, University of Rome “Tor Vergata,” Rome; 2 Department of Cellular Biotechnologies and Hematology, Faculty of Pharmacy and Medicine, Sapienza University of Rome, Rome; 3 Pasteur Institute-Fondazione Cenci Bolognetti, Rome; 4 National Institute of Health and Science on Aging (INRCA), Nutrition and Ageing Centre, Scientific and Technological Research Area, Ancona; 5 IRCCS Istituto delle Scienze Neurologiche di Bologna, Bologna; 6 Department of Experimental, Diagnostic and Specialty Medicine, Bologna, Italy; 7 CIG-Interdepartmental Center “L. Galvani,” Alma Mater Studiorum, University of Bologna, Bologna, Italy; 8 Institute of Nutritional Medicine (180c), University of Hohenheim, Stuttgart; 9 German Institute of Human Nutrition Potsdam-Rehbruecke (DIfE), Nuthetal; 10 Department of Biology, Molecular Toxicology Group, University of Konstanz, Konstanz, Germany

**Keywords:** 5-hydroxymethylcytosine, TET enzymes, DNA methylation, aging

## Abstract

Down syndrome (DS) is caused by the presence of part or an entire extra copy of chromosome 21, a phenomenon that can cause a wide spectrum of clinically defined phenotypes of the disease. Most of the clinical signs of DS are typical of the aging process including dysregulation of immune system. Beyond the causative genetic defect, DS persons display epigenetic alterations, particularly aberrant DNA methylation patterns that can contribute to the heterogeneity of the disease. In the present work, we investigated the levels of 5-hydroxymethylcytosine and of the Ten-eleven translocation dioxygenase enzymes, which are involved in DNA demethylation processes and are often deregulated in pathological conditions as well as in aging. Analyses were carried out on peripheral blood mononuclear cells of DS volunteers enrolled in the context of the MARK-AGE study, a large-scale cross-sectional population study with subjects representing the general population in eight European countries. We observed a decrease in 5-hydroxymethylcytosine, *TET1*, and other components of the DNA methylation/demethylation machinery in DS subjects, indicating that aberrant DNA methylation patterns in DS, which may have consequences on the transcriptional status of immune cells, may be due to a global disturbance of methylation control in DS.

Down Syndrome (DS) is the most common genetic autosomal disorder compatible with life in humans. In the majority of cases, DS is a whole-chromosome trisomy due to meiotic nondisjunction of chromosome 21. The phenotypical features of the disease are multiple and heterogeneous, and they mainly comprise intellectual disability, growth defects, and endocrine disorders. Moreover, DS individuals are characterized by an acceleration of the aging process, affecting particularly the immune and central nervous system. In fact, individuals with DS show alterations of innate and adaptive immunity cell subsets and are more prone to develop dementia and Alzheimer’s disease (1–3). Accordingly, we recently demonstrated that neuropsychological functions and adaptive skills show an age-associated decline in persons affected by DS, including those recruited in the MARK-AGE study and further studied in this work (4,5). Moreover, an accelerated aging was also revealed in DS when N-glycans were analyzed (6).

Recently, much effort has been invested into the investigation of epigenetic signatures in DS persons (7,8). Attention has mainly been focused on DNA methylation for its prominent role in the control of gene expression both in physiological and pathological conditions. In mammals, DNA methylation originates from the addition of a methyl group to cytosine bases, generally located at the CpG dinucleotides, by the DNA methyltransferase (DNMT) family enzymes (DNMT1, DNMT3A, and DNMT3B) to form 5-methylcytosine (5mC). DNA methylation patterns are established and maintained to define transcriptional networks associated with cell-type identity. Several proteins are involved in the regulation DNA methylation dynamics including chromatin readers of 5mC, methylation-sensitive transcription factors, and, particularly, the Ten-eleven translocation (TET) family of DNA hydroxylases. In fact, TET enzymes (TET1, TET2 and TET3) catalyze the oxidation of 5mC to 5-hydroxymethylcytosine (5hmC), 5-formylcytosine (5fC), and 5-carboxylcytosine (5caC), which can act as distinct epigenetic marks or represent intermediates of DNA demethylation. In particular, TET-mediated active DNA demethylation entails the thymine DNA glycosylase (TDG)-mediated DNA repair pathway for the removal of unnecessary oxidized 5mC (9).

A number of epigenome-wide analyses have shown differences in DNA methylation patterns between DS and control persons in brain, blood, buccal epithelial cells, and extra-embryonic tissues (8,10,11). These studies unambiguously demonstrated that in DS aberrantly methylated regions are distributed across all chromosomes and not limited to chromosome 21. Both hypermethylation and hypomethylation events were found in DS samples, although the incidence of hypermethylation was generally higher in brain regions and placenta (8,10,11). The accelerated aging phenotype of DS is mirrored by epigenetic alterations, as the disease increases DNA methylation age in whole blood (on average, 4.6 years), in leukocytes (on average, 3.9 years), and in brain (on average, 11.5 years) (12). Moreover, DNA methylation changes were already detectable in fetal and early postnatal development (8).

The contribution of 5hmC in DS pathology, however, is less characterized. Down-regulation of *TET* genes was suggested to be associated with DNA hypermethylation in placenta from DS samples (11), whereas hypomethylation of a *TET1* gene regulative region (ie *TET1* CpG island 3′-shore) was demonstrated to occur in whole blood from DS persons compared with their siblings (10). Only recently, a report has highlighted that 5mC and 5hmC additively account for most of the alterations of DNA methylation in DS cerebellum. A part of these alterations, however, were at the expense of 5hmC only; in that case, the levels of 5hmC were mainly decreased in DS samples (8). Notably, global changes in 5hmC levels have been identified in several neurodegenerative conditions (eg Alzheimer’s disease and multiple sclerosis), and loss of 5hmC is considered a hallmark of cancer. Moreover, we and others have recently demonstrated that blood cells undergo a gradual loss of 5hmC in aging (13,14). In particular, we demonstrated that the reduction of 5hmC was accompanied by decreased expression of *TET1* and *TET3* (14). Our results were obtained on peripheral blood mononuclear cells (PBMCs) deriving from volunteers enrolled in the Europe-wide cross-sectional population study “MARK-AGE.” In this context, also a cohort of DS persons was recruited because DS shares similarities with progeroid diseases, due to the precocious occurrence of age-associated features (1,3). In order to assess the role of DNA hydroxymethylation in DS immune cells we analyzed 5hmC levels and the expression of *TET* genes in PBMCs from DS persons compared with control individuals.

## Materials and Methods

### Study Population, Recruitment, Data, and Blood Collection

PBMC samples used in the present work derived from an Italian cohort of DS blood donors (*n* = 50; age mean = 40 ± 12; 48% female) and control volunteers from Italy (*n* = 45; age mean = 49 ± 12; 43% female) that have been recruited in the context of the MARK-AGE project (5,15,16). A group of young (*n* = 52; age mean = 38 ± 4; 58% female) individuals and another one of elders (*n* = 49; age mean = 78 ± 2; 45% female), enrolled in the same project from different European countries and previously tested (14), were also used for comparison with DS samples. The recruitment procedure and PBMC isolation details have been published (17,18). Briefly, PBMCs were isolated from EDTA-whole blood, obtained by phlebotomy after an overnight fasting, by discontinuous density gradient centrifugation in Percoll. Isolated cells were subsequently cryopreserved, stored in liquid nitrogen, and shipped to the MARK-AGE Biobank located at the University of Hohenheim, Stuttgart, Germany. From the Biobank, coded samples were subsequently sent to the Sapienza University of Rome on dry ice, where they were stored in liquid nitrogen.

### RNA Extraction and cDNA Synthesis

Samples were thawed by incubation at 37°C, followed by dropwise addition of RPMI containing 10% FCS to a final dilution of 1:20. After centrifugation, cells were processed for RNA extraction by using RNeasy Mini Kit (Qiagen) according to the manufacturer’s instructions and subjected to DNase I digestion using RNase-free DNase (Qiagen). RNA concentration, purity, and integrity were evaluated as previously described (19). Reverse transcription was carried out using the SuperScript VILO cDNA Synthesis Kit (Life technologies) on equal amounts of total RNA.

### Real-Time Quantitative RT-PCR

The expression of candidate genes was determined by quantitative PCR using Taqman Gene Expression Assays (Applied Biosystems) following the manufacturer’s protocol on the iCycler IQ detection system (Bio-Rad). Each set of primers and probe showed an efficiency of 90%–100%. Assays were performed in duplicate with 30 ng of reverse transcribed RNA. Gene expression analysis was performed by the relative calibrator normalized quantification method using the expression level of the β-glucuronidase gene (*GUSB*) as reference (19). An inter-run calibration sample was used in each plate to correct for technical variance between runs and to compare results from different plates. The calibrator consisted of cDNA prepared from HEK293T cells. Taqman Gene Expression Assays IDs for each set of primers and probe were as follows: Hs00286756_m1 (*TET1*); Hs00758658_m1 (*TET2*); Hs00379125_m1 (*TET3*); Hs00702322_s1 (*TDG*); Hs00154749_m1 (*DNMT1*); Hs01027166_m1 (*DNMT3A*); Hs00171876_m1 (*DNMT3B*); and Hs99999908_m1 (*GUSB*).

### Dot Blot Assay

DNA was extracted from PBMCs with DNeasy Blood & Tissue Kit (QIAGEN), denatured in 0.4 M NaOH, 10 mM EDTA at 95°C for 10 minutes, and then neutralized by adding an equal volume of ice-cold 4 M ammonium acetate (pH 7.0). Two-fold dilutions of DNA samples (250 ng) were spotted on the positively charged nylon membranes Hybond-N+ (Amersham Biosciences) in an assembled Bio-Dot apparatus (Bio-Rad). After vacuum-mediated blotting, the membrane was washed with 2× SSC buffer, air-dried, and blocked with 5% nonfat dry milk followed by incubation with primary polyclonal anti-5hmC antibody (Active Motif). After incubation with HRP-conjugated secondary antibody, signals were visualized by chemiluminescence (Amersham ECL Western Blotting detection reagents). To correct for technical variance between replicates and to compare results from different experiments, control DNA obtained from HEK293T cells was used in all assays. Filters were stained with 0.02% methylene blue (MB) in 0.3 M sodium acetate (pH 5.2) to monitor DNA loading. Densitometric analysis was performed by Quantity One Software (Bio-Rad Laboratories) according to manufacturer’s instructions.

### EpiTYPER Assay for Quantitative DNA Methylation Analysis

The EpiTYPER assay (Sequenom) was used to quantitatively assess the DNA methylation state of *TET1* CpG island. DNA (1 μg) was bisulfite-converted using the EZ-96 DNA Methylation Kit (Zymo Research) with the following modifications: incubation in CT buffer for 21 cycles of 15 minutes at 55°C and 30 seconds at 95°C, elution of bisulfite-treated DNA in 100 μl of water. PCR was performed on 10 ng of converted DNA using *TET1* CGI_Fw: aggaagagagGGTTTTTAGTTTTAA GTTTGTATTAGTTTT and *TET1* CGI_Rev: cagtaatacgactcactataGGGAGAAGGC TATCATACAACCCTACCTACCTCTCC primers.

### Statistical Analysis

For continuous variables, normal distribution of data was verified by Kolmogorov–Smirnov and Shapiro–Wilk normality tests (data not shown). Log transformation of data was used, as some variables were not normally distributed. When log-transformed data displayed normal distribution parametric tests were applied, otherwise, we used nonparametric tests. In particular, the identification of potential critical variables that can affect the variable under examination was performed by Mann–Whitney U-test and Student’s *t*-test for two-group comparisons. One-way ANOVA followed by Tukey’s post hoc test was used for more than two independent groups. When a significant *p*-value was found, a multiple comparison test by generalized linear models (GLMs) was performed and pairwise comparisons (adjusted for multiple comparisons by Dunn’s and Bonferroni’s methods for the Kruskal–Wallis and the GLM tests, respectively) were used to identify significant differences between percentile groups of each categorized variable. The Bonferroni adjustment for multiple comparisons was used to confirm differences between subgroups. GLMs were also used to investigate the influence of confounding variables (tested as categorized and continuous variables) on group-related changes of analyzed parameters. Pearson correlations were used to assess associations between defined variables. All statistical analyses were carried out using SPSS software (SPSS Inc., Chicago, IL; Version 22.0).

## Results

### Analysis of 5hmC Levels in PBMCs From DS Samples

**Figure 1.**
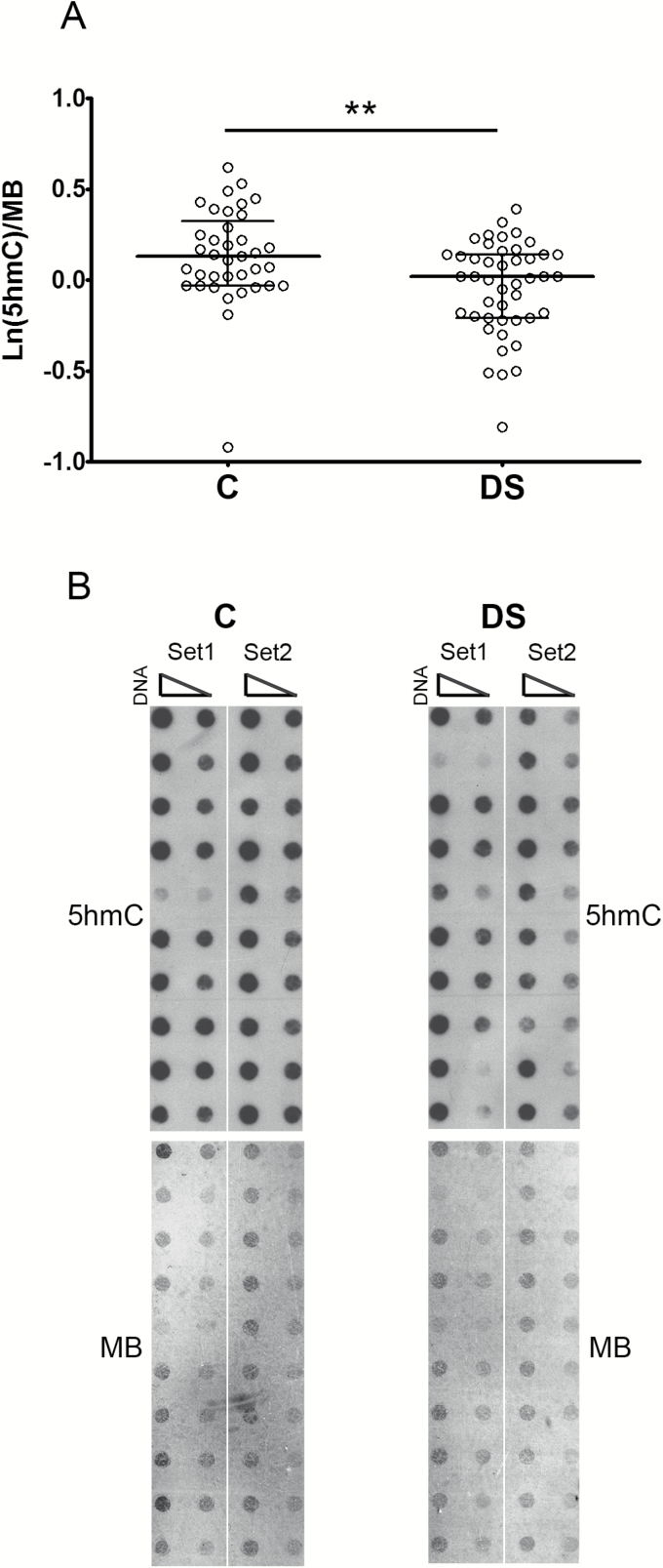
5-hydroxymethylcytosine levels are decreased in DS PBMCs. (**A**) Scatter plot showing median and interquartile ranges of 5hmC levels in DS and control (C) subjects. Statistical analysis was performed by Mann–Whitney U-test. (**B**) Representative dot blot of DNA from DS and control subjects performed by using anti-5hmC antibody (left panel) and methylene blue (MB) staining (right panel) as a DNA loading control, **p < .01.

The content of 5hmC was measured by dot-bot assay on DNA from PBMCs obtained from DS persons and healthy controls. As shown in Figure 1, the levels of 5hmC are lower in DS samples compared with controls, as determined by Mann–Whitney U-test (*p* = .0037). To determine the influence of the covariates gender, age, and leukocyte composition (lymphocyte/monocyte ratio) on the 5hmC levels, the multivariate tests and the GLM procedures were computed (Supplementary Tables 1 and 2). These analyses demonstrated that the difference in 5hmC content between DS and controls was independent of any of those variables.

### Expression of *TET* Genes in PBMCs From DS Samples

**Figure 2.**
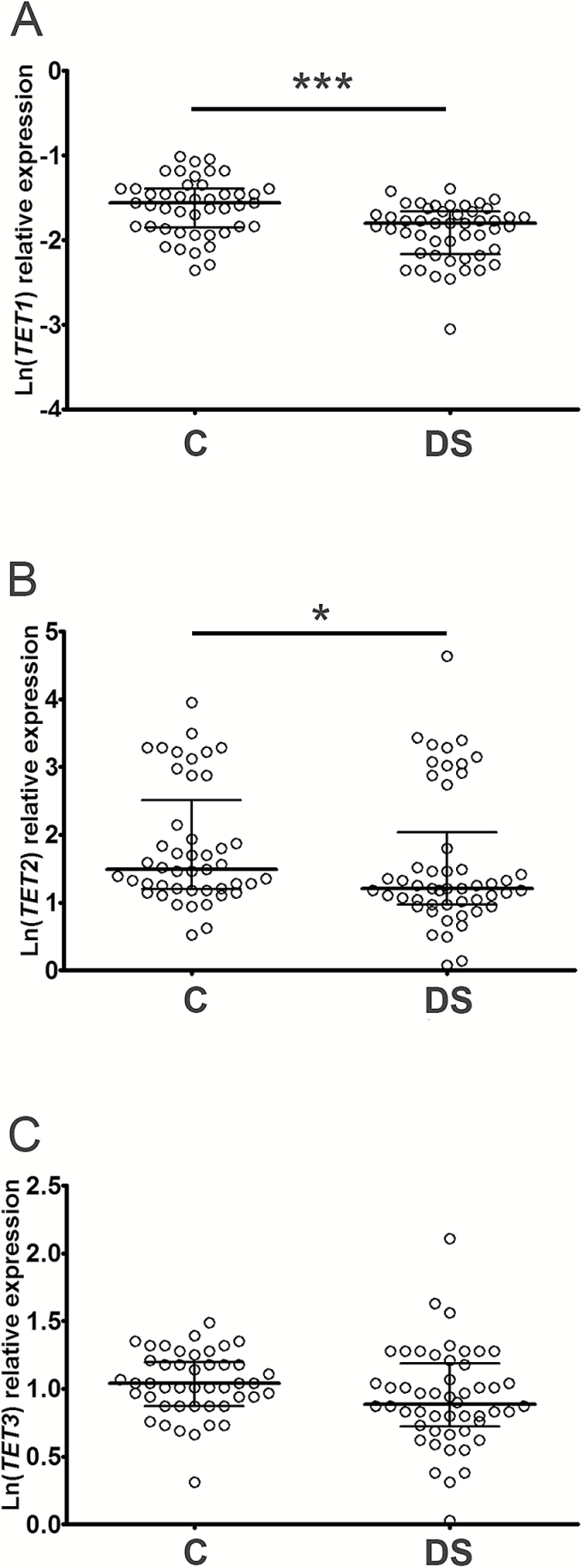
*TET1* expression levels are decreased in DS PBMCs. Scatter dot plot showing median and interquartile ranges of *TET1* (**A**), *TET2* (**B**), and *TET3* (**C**) mRNA levels in DS and control (C) subjects. Statistical analysis was performed by unpaired *t*-test for *TET1* and Mann–Whitney U-test for *TET2,* *p < .05; ***p < .001.

Subsequently, analyses were performed to see if the reduction of 5hmC levels in DS samples was associated with a deregulation of the enzymes involved in 5hmC formation. Expression of the 5mC-hydroxylase enzymes *TET1, TET2*, and *TET3* was assessed by the real-time PCR analyses demonstrating that the levels of *TET1* and *TET2* were reduced in DS samples with respect to controls (*TET1 t*-test: *p* < .001; *TET2* Mann–Whitney U-test: *p* = .0295; Figure 2). However, multivariate test analysis demonstrated that the expression of *TET2* was affected by confounding factors while *TET1* expression was independent of age, gender and lymphocyte/monocyte ratio, as also highlighted by GLM analysis (Supplementary Tables 1 and 3). The significant decrease in *TET1* expression in DS sample was also confirmed by a generalized mixed linear model introducing batch effects as random factor (Supplementary Table 4). As concerns *TET3*, its expression was slightly reduced in DS samples, but this result was not statistically significant (*TET3 t*-test: *p* = .0926; Figure 2) and affected by covariates (Supplementary Table 5).

We then analyzed the correlation between 5hmC levels and expression of *TET* genes demonstrating a positive association with *TET1* (*r* = 0.2729, *p* = .0131) and also a positive trend, albeit not significant, with *TET2* (*r* = 0.1959, *p* = .0778; Supplementary Figure 1). A possible coregulation between *TET* enzymes was also tested by correlation analysis and a relevant association of *TET2* with *TET3* was observed (*r* = 0.3595, *p* = .0003), but not of *TET1* with both of them (*r*_(*TET2*)_ = 0.131, *p* = .2081; *r*_(*TET3*)_= −0.0413, *p* = .6711).

### DNA Methylation Analysis of *TET1* CpG Island

**Figure 3.**
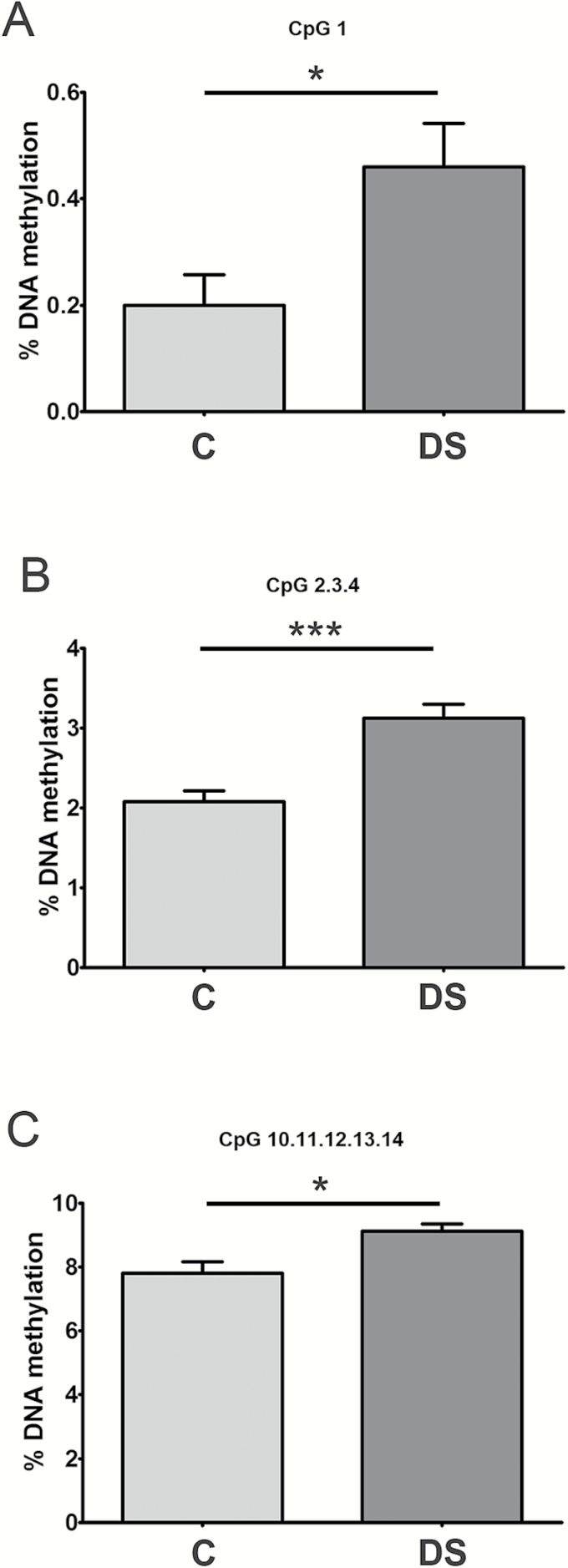
DNA methylation levels at the *TET1* CpG island in DS and control PBMCs. Graphs represent the CpGs at *TET1* CpG island showing difference in DNA methylation between DS and control (C) subjects. DNA methylation analysis was performed by epiTYPER assay. Statistical significance was determined by unpaired *t*-test, *p < .05; ***p < .001.

To investigate whether DNA methylation is responsible of *TET1* down-regulation in PBMCs from DS persons, quantitative analysis of DNA methylation and identification of differentially methylated CpG sites at the CpG island of *TET1* gene were assessed by using MassARRAY EpiTYPER. The analysis was performed with primers encompassing a 215-bp DNA region mapped to chr10:70,320,251-70,320,466. The comparison of the epigenetic profile between DS and control healthy individuals demonstrated that few CpGs present at the *TET1* CpG island were slightly but significantly hypermethylated in DS PBMC samples (Figure 3 and Supplementary Figure 2A). As some of those CpGs were also shown to undergo hypermethylation with aging, we compared the DNA methylation levels at *TET1* CpG island in DS with a group of elderly population (age range: 69–74 years), and in this case, no difference was observed at any CpG (Supplementary Figure 2B).

### Expression of *DNMT*s and *TDG* in PBMCs From DS Samples

**Figure 4.**
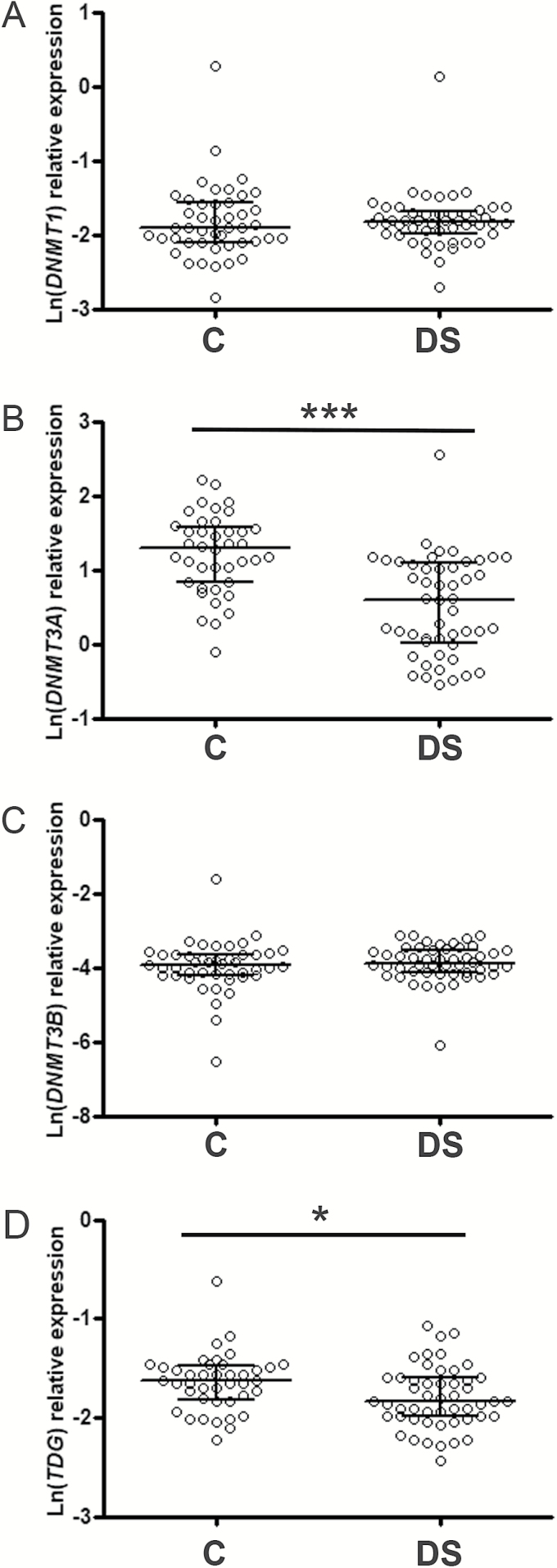
The expression levels of *DNMT3A* and *TDG* are decreased in DS PBMCs. Scatter plot showing median and interquartile ranges of *DNMT1* (**A**), *DNMT3A* (**B**), *DNMT3B* (**C**), and *TDG* (**D**) levels in DS and control (C) subjects. Statistical analysis was performed by Mann–Whitney U-test for *DNMT3A* and unpaired *t*-test for *TDG*, *p < .05; ***p < .001.

To better characterize changes in relevant epigenetic enzymes connected with 5hmC in DS samples, the expression of the active DNA methyltransferase enzymes (*DNMT1*, *DNMT3A*, and *DNMT3B*) and of *TDG*, the main glycosylase involved in TET-mediated DNA demethylation, was also assessed. Results demonstrated that the levels of *DNMT1* and *DNMT3B* mRNA were similar between DS and control samples (*DNMT1* Mann–Whitney U-test: *p* = .4808; *DNMT3B* Mann–Whitney U-test: *p* = .2829), whereas *DNMT3A* and *TDG* genes were down-regulated in PBMCs from DS persons (*DNMT3A* Mann–Whitney U-test: *p* < .0001; *TDG t*-test: *p* = .0136; Figure 4). These differences were confirmed by multivariate tests and GLM analysis (Supplementary Tables 1, 6, and 7), which also highlighted that the expression of *DNMT3A* was partially influenced by the leukocyte composition of PBMCs (Supplementary Table 6). In particular, the impact of lymphocyte/monocyte ratio on *DNMT3A* expression was mainly ascribable to control individuals, as a relevant positive correlation was observed in them (*r* = 0.4710, *p* = .0033) but not in DS persons (*r* = −0.01432, *p* = .9222; Supplementary Figure 3). In addition, due to the clear-cut reduction of *DNMT3A* in DS, we tested a possible association with the levels of 5hmC and *TET* genes demonstrating a positive correlation with *TET2* (*r* = 0.2302, *p* = .03) and *TET3* (*r* = 0.3895, *p* = .0002) but not with *TET1* (*r* = 0.1259, *p* = .2396) and 5hmC (*r* = 0.07261, *p* = .5275; Supplementary Figure 4).

### Analysis of 5hmC and DNA Methylation/Demethylation Machinery in DS, Young Persons, and Elders

**Figure 5.**
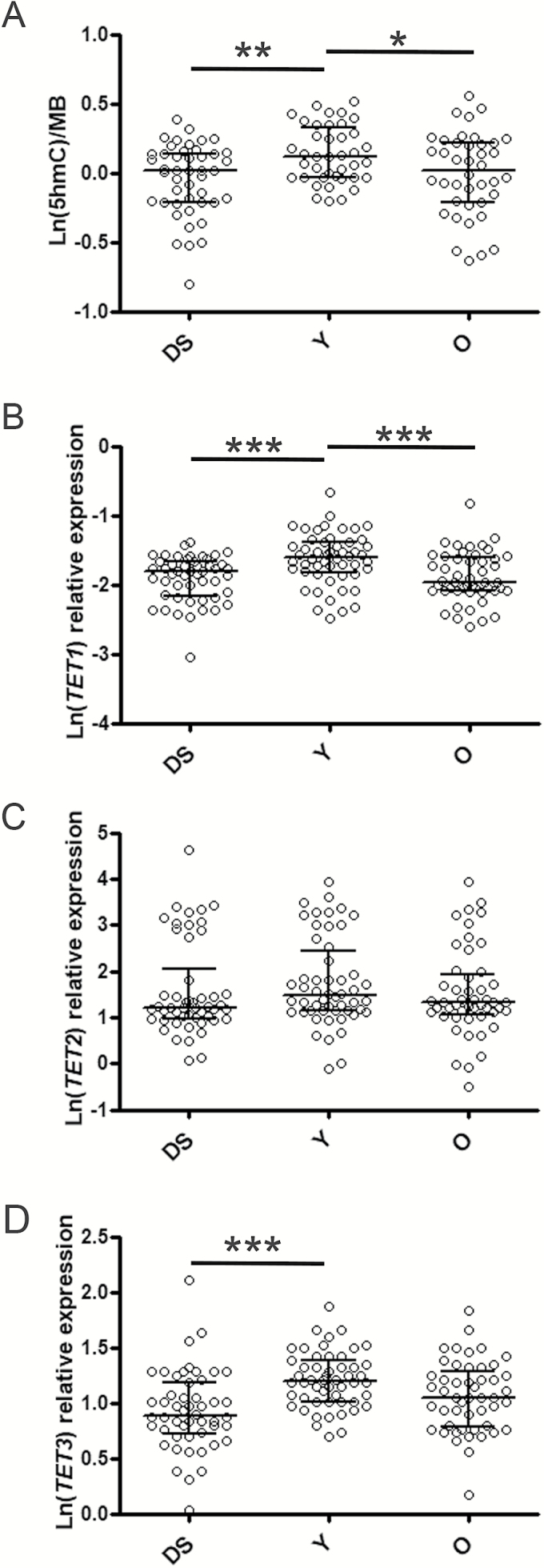
5hmC levels as well as *TET1* and *TET3* expression were similar between DS samples and an elderly population. Scatter plot showing median and interquartile ranges of 5hmC (**A**), *TET1* (**B**), *TET2* (**C**), and *TET3* (**D**) levels in DS persons, young (Y) persons (age: 31–45 years) and old (O) persons (age: 69–74 years). Statistical analysis was performed by one-way ANOVA with post hoc Tukey’s test, *p < .05; **p < .01; ***p < .001.

As DS is considered a disease with premature aging features, we decided to compare the variables so far analyzed in DS with groups of young (age range: 31–45 years) and elderly people (age range: 69–74 years) previously assessed (14). Notably, we observed that levels of 5hmC, *TET1*, and *TET3* in DS were significantly lower than young persons but not different from the elderly group (Figure 5), also when considering gender, country of origin and leukocyte composition as covariates in GLM analysis (Supplementary Tables 8–10). On the contrary, *DNMT1* expression was lower in the elderly population than both DS and young persons (Supplementary Figure 5), but GLM analysis did not confirm the result due to a relevant influence of recruitment center (Supplementary Table 11). A difference between groups was obtained for *TDG* by GLM analysis independently of covariates but the pairwise comparison was statistically significant only for young and old people groups (Supplementary Figure 5 and Supplementary Table 12). No change was observed for *TET2* and *DNMT3B* expression (Figure 5, Supplementary Figure 5, and Supplementary Tables 13 and 14), whereas *DNMT3A* was not tested as no data were available for the young and old populations. Moreover, it is noteworthy that most of variables (ie 5hmC, *TET1*, *TET3*, *TDG*, and *DNMT1*) showed a negative correlation with age when considering young and old people together, but this association was lost when DS persons were included. Only the negative correlation of *DNMT1* with aging was retained when all samples were grouped (Supplementary Table 15).

## Discussion

The presence of an extra-copy of genes located in chromosome 21 has long been considered the only driving cause for the DS pathogenesis. However, DS does not only originate from aneuploidy of the entire or fragmented chromosome 21 itself, but different DNA regions have been identified to be affected by trisomy and associated with different phenotypes. Along with the gene dosage theory, studies on both murine and human models have suggested that several other mechanisms determine the DS phenotype. In fact, the complexity of DS also depends on the *trans* effects that each karyotype can induce on distal genomic loci by physical interactions due to higher-order structure of chromatin. Moreover, much importance has been attributed to epigenetics in the pathogenesis of DS as numerous papers have highlighted *trans* effects of trisomy 21 also on DNA methylation patterns (7,8). Regions that are differentially methylated in healthy and DS individuals are largely distributed along the entire genome in several tissues. Most of the DNA methylation differences are tissue specific, but a certain degree of overlap has been shown in brain and T-cells and largely corresponding to specific transcription factor binding sites (8).

The contribution of 5hmC in DS was investigated in brain (8), the tissue with the highest levels of DNA hydroxymethylation, in which differentially hydroxymethylated regions were observed in genes largely related to brain development and function. In addition, hyperhydroxymethylation has been shown to characterize the internal promoter region of the PR domain containing 8 (*PRDM8*) gene in PBMCs of DS samples, in which an up-regulation of the associated transcript variant 2 was observed (20).

Our present work is the first one describing alterations of 5hmC in blood cells from DS persons. In particular, we provide evidence of decreased 5hmC levels in DS, an event frequently occurring in many multifactorial pathological conditions, such as cancer and neurodegeneration (21,22). Notably, 5hmC reduction has also been observed in another chromosomal disorder known as constitutional trisomy 8 mosaicism (23). The drop of 5hmC in DS samples is mainly associated with the down-regulation of *TET1*. Moreover, we show hypermethylation of few CpGs at *TET1* promoter in DS samples when compared with age-matched controls, but this slight increase in 5mC is unlikely to explain by itself the silencing of *TET1*. Notably, we observed no difference with the methylation profile of a group of elderly population enrolled in the context of the same project (14). This result together with the evidence that 5hmC, *TET1*, and *TET3* in DS show similar level of the elderly population and lower than the young one supports the view of DS as a disorder characterized by premature aging features.

Alteration of DNA methylation is by now a well-established phenomenon occurring in DS (8,24), but, at least in blood, no information exists about the levels of DNMT enzymes. Our analysis of the transcripts of the active methyltransferases demonstrated a down-regulation of *DNMT3A* in DS PBMCs. A reduction of *DNMT3A* expression along with *DNMT3B* has been recently reported in DS brain (25), whereas no difference for *DNMT3A* was observed in induced pluripotent stem cells generated from the twins discordant for DS (26). In addition, deregulation of DNMT3A as well as of other DNMTs has been documented in several tissues during aging (27–29).

Among the clinical manifestations typical of both aging and DS, 5hmC and *TET* deregulation in PBMCs may contribute to the decline of immune function, which is associated with increased susceptibility to infection and autoimmune diseases. New-borns with DS already show an abnormal structure of thymus, the central organ of T lymphocytes differentiation, with a concomitantly decreased production of mature T cells in the periphery (30). Notably, dynamic changes of 5hmC have been characterized during development and differentiation of T-cell lineage. Thymus-specific enhancers and gene bodies of highly transcribed genes showed enrichment of 5hmC. Moreover, higher levels of 5hmC were detected in active genes of precursor cells, suggesting the functional importance of 5hmC in T-cell specification (31). 5hmC-driven active demethylation has also been implicated in the thymic development of regulatory T cells (Tregs), a small subpopulation of T cells with fundamental roles in immune homeostasis control and thus in the suppression of autoimmunity. In fact, TET1 and TET2 activities were demonstrated to mediate the demethylation-dependent activation of *FOXP3* gene, the master regulator of Tregs development and function (32,33). In addition, DS persons also have a significantly higher risk of developing leukemia, particularly acute lymphoblastic leukemia and acute megakaryoblastic leukemia (34), the onset of which has been shown to be accompanied by sequential epigenetic changes in DS (35). Notably, loss of 5hmC is a typical feature of blood cell malignancies and of solid tumors as well (36). TET1 expression is frequently deregulated in solid tumors, but a prominent role in the regulation of B-cell malignancies has been demonstrated, and evidence of its mutations exists in T-acute lymphoblastic leukemia (37–39). TET2 is a master regulator of normal hematopoiesis, and mutations of the *TET2* gene have been frequently identified in multiple spectra of myeloid malignancies (40,41). Similarly, among DNMT enzymes, *DNMT3A* represents one of the most frequently mutated genes in diverse types of hematological cancers (41). Finally, it is worth noting that *DNMT3A* and *TET2* mutations are also frequently observed in age-related clonal hematopoiesis (42), suggesting a central role of these two enzymes in age-related phenotypes and tumor development.

In conclusion, our analysis of DNA hydroxymethylation in PBMCs from DS patients demonstrated a decrease in 5hmC levels and components of the relative enzymatic machinery. The positive correlation between *TET1* expression and 5hmC levels may suggest a causative role of *TET1* down-regulation in DNA hypermethylation in DS patients. Viewed together, our results further strengthen the evidence that multiple pathological states are associated with deregulation of 5hmC that can contribute to the establishment of disease-associated DNA methylation patterns. Apart from being an intermediate in DNA demethylation processes, 5hmC directly controls gene expression programs acting as an epigenetic mark in its own right, recognized by specific chromatin readers. Therefore, the epigenetic consequences downstream of 5hmC alteration in blood cells can induce age-related clinical impairment of DS immune system (eg immunosenescence), with higher occurrence of infections, hematological malignancies, and autoimmune diseases in DS persons.

## Supplementary Material

Supplementary data is available at *The Journals of Gerontology, Series A: Biological Sciences and Medical Sciences* online.

## Funding

This work was supported by the European Union’s Seventh Framework Program [grant HEALTH-F4-2008–200880 MARK-AGE].

## Conflicts of Interest Statement

None declared.

## Supplementary Material

Supplementary_Information
