# Supplementary material for: DNA Hydroxymethylation Levels Are Altered in Blood Cells From Down Syndrome Persons Enrolled in the MARK-AGE Project
Source: J Gerontol A Biol Sci Med Sci. 2017 Oct 21;73(6):737–44. doi: 10.1093/gerona/glx198 (PMC5946825; doi:10.1093/gerona/glx198)
Supplement: Supplementary_Information [file glx198_suppl_supplementary_information.docx]

**Supplementary Materials**

**

**

**Supplementary Figure 1: Positive correlation between 5hmC levels and *TET1* gene expression.** The linear association between 5hmC and *TET1* (**A**), *TET2* (**B**), *TET3* (**C**) expression levels was calculated using parametric (Pearson r) correlation coefficients based on Ln-transformed data.

A







B

**Supplementary Figure 2. DS samples show hypermethylation of CpGs at *TET1*** **CpG island when compared to age-matched control individuals but not to an old population group.** DNA methylation levels of the CpG island of *TET1* assessed by Sequenom EpiTYPER in PBMC of DS compared to: **A**) a group of age-matched control (C) individuals and **B**) a group of elderly people (O) with age years 69-74. Statistical significance was analysed by unpaired t-Test (*p < 0.05; ***p < 0.001).


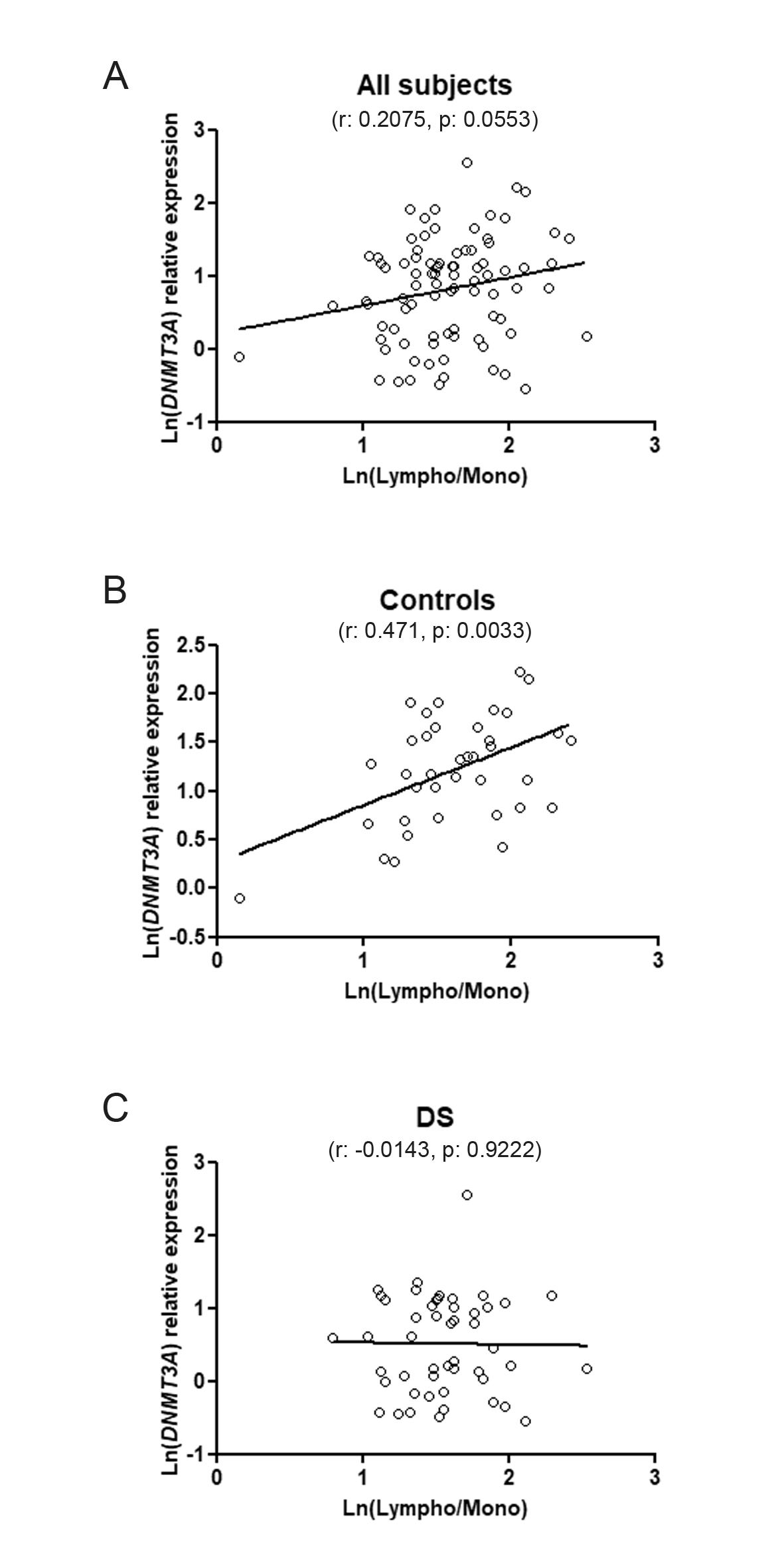







**Supplementary Figure 3: Correlation analysis between *DNMT3A* levels and lymphocyte to monocyte ratio.** The linear association between *DNMT3A* expression levels and leukocyte composition in terms of lympho/mono ratio was calculated using parametric (Pearson r) correlation coefficients based on Ln-transformed data on the whole population of DS and Controls (**A**) and on Controls (**B**) or DS (**C**) samples alone.







**Supplementary Figure 4: Correlation analysis between *DNMT3A* expression and the levels of 5hmC and *TET*s.** The parametric (Pearson r) correlation coefficients based on Ln-transformed data were calculated on the whole population between *DNMT3A* expression and 5hmC (**A**), *TET1* (**B**), *TET2* (**C**) and *TET3* (**D**) levels.


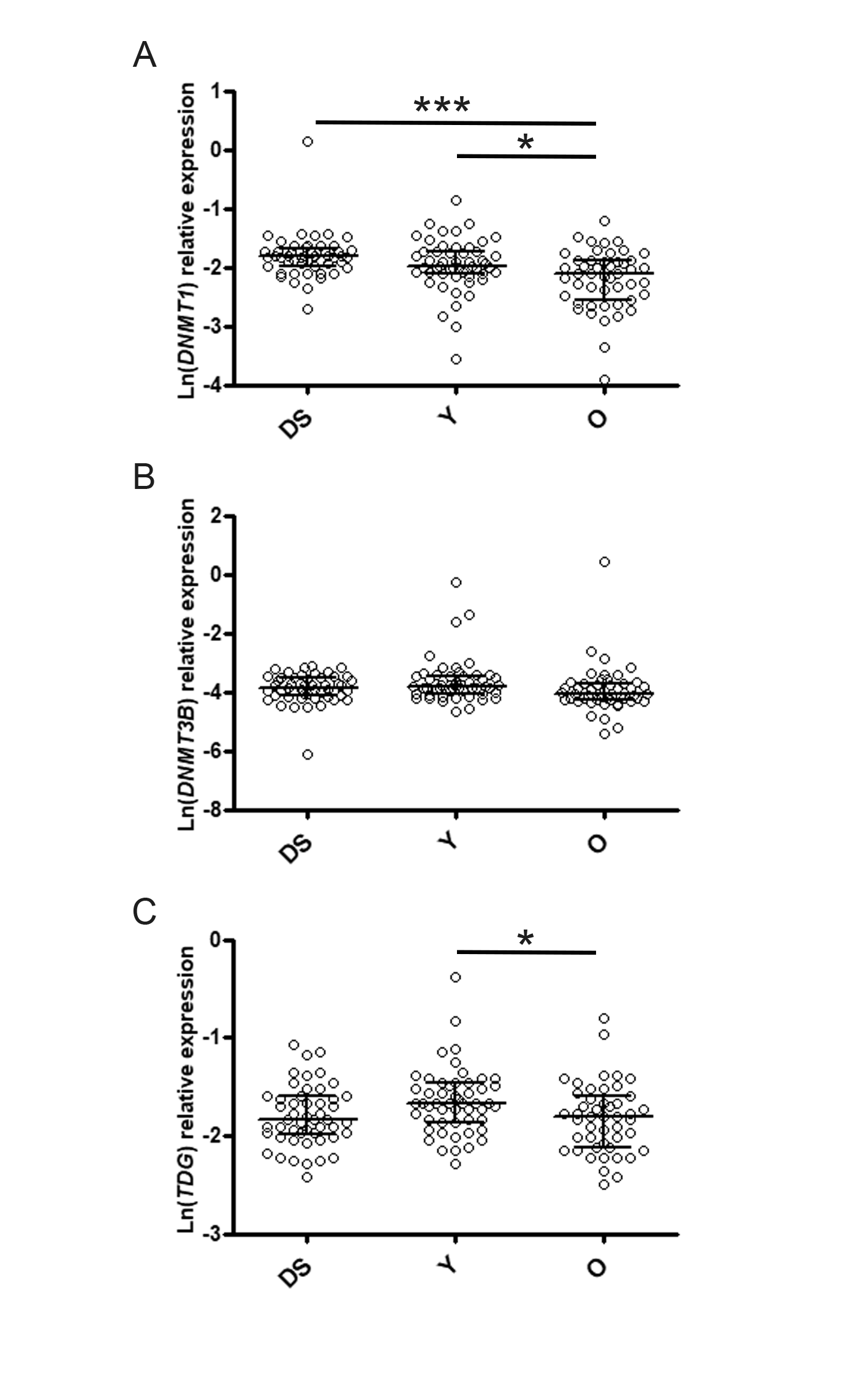


**Supplementary Figure 5: Comparison of *DNMT*s and *TDG* levels between DS, young and old persons.** Difference in expression levels of *DNMT1* (**A**), *DNMT3B* (**B**) and *TDG* (**C**) was assessed in DS persons, young (Y) persons (age years 31-45) and old (O) persons (age years 69-74) by performing One-way ANOVA with post-hoc Tukey test. (*p < 0.05; ***p < 0.001).

**Supplementary Table 1.** Explorative analysis by multivariate tests for assessing the effect of selected covariates on variables

| ***Multivariate Tests^a^*** | | | ***Test of between-subjects effects***  ***for Subject groups (DS vs control)*** | | |
| --- | --- | --- | --- | --- | --- |
| **Effect** | **F** | **p** | **Variables** | **F** | **p** |
|  |  |  | **Ln(*TET1*)** | 14.454 | < 0.001 |
| **Intercept** | 46.143 | < 0.001 | **Ln(*TET2*)** | 1.703 | 0.196 |
| **Age** | 0.964 | 0.473 | **Ln(*TET3*)** | 6.013 | 0.017 |
| **Ln(lympho/mono)** | 2.959 | 0.007 | **Ln(*TDG*)** | 5.333 | 0.024 |
| **Subject groups** | 7.077 | < 0.001 | **Ln(5hmC)** | 7.029 | 0.010 |
| **Gender** | 1.576 | 0.152 | **Ln(*DNMT1*)** | 0.929 | 0.338 |
| **Subject groups*gender** | 1.083 | 0.388 | **Ln(*DNMT3B*)** | 1.225 | 0.272 |
|  |  |  | **Ln(*DNMT3A*)** | 24.224 | < 0.001 |
|  |  |  |  |  |  |

*^a^* Design: Intercept + Age + Ln(lympho/mono) + Subject groups + gender + Subject groups*gender. F values and significance were equal for Pillai’s Trace, Wilks’ Lambda, Hotelling’s Trace, Roy’s Largest Root tests. Dependent variables: Ln(*TET1*), Ln(*TET2*), Ln(*TET3*), Ln(*TDG*), Ln(5hmC), Ln(*DNMT1*), Ln(*DNMT3B*), Ln(*DNMT3A*). Subject groups: Control and DS.

**Supplementary Table 2.** Contribution of selected variables and covariates on 5hmC variation across control and DS subject groups †

*Test of Model Effects* **Ln(5hmC)**

|  | **Type III** | | |
| --- | --- | --- | --- |
| Variables | **Wald**  **Chi-Square** | **df** | **p** |
|  |  |  |  |
| **(Intercept)** | 0.986 | 1 | 0.321 |
| **Subject groups** | 9.128 | 1 | 0.003 |
| **Gender** | 1.163 | 1 | 0.281 |
| **Age** | 1.271 | 1 | 0.26 |
| **Ln(lympho/mono)** | 0.01 | 1 | 0.92 |

†Analysis was performed by GLM using linear model with identity link function considering as dependent variable: Ln(5hmC). Model: (Intercept), Subject groups (Healthy and DS), gender, age (years), Ln(lymphocyte/monocyte).

**Supplementary Table 3.** Contribution of selected variables and covariates on *TET1* variation across control and DS subject groups †

*Test of Model Effects* **Ln(*TET1*)**

|  | **Type III** | | |
| --- | --- | --- | --- |
| Variables | **Wald**  **Chi-Square** | **df** | **p** |
|  |  |  |  |
| **(Intercept)** | 56.227 | 1 | < 0.001 |
| **Subject groups** | 15.919 | 1 | < 0.001 |
| **Gender** | 0.002 | 1 | 0.965 |
| **Age** | 0.517 | 1 | 0.472 |
| **Ln(lympho/mono)** | 3.444 | 1 | 0.063 |

†Analysis was performed by GLM using linear model with identity link function considering as dependent variable: Ln(*TET1*). Model: (Intercept), Subject groups (Control and DS), gender, age (years), Ln(lymphocyte/monocyte)

**Supplementary Table 4.** GLM analysis with random effects to verify the robustness of *TET1* difference between Control and DS subjects†

*Model Effects*

Fixed coefficient **Ln(*TET1*)**

|  |  | | |
| --- | --- | --- | --- |
| Model Term | **Coefficient** |  | **p** |
|  |  |  |  |
| **Intercept** | 0.2 |  | 0.000 |
| **Subject groups** | -0.057 |  | 0.007 |
| **Lympho/mono** | 0.008 |  | 0.007 |

†Analysis was performed by GLM using linear model with identity link function considering as dependent variable: Ln(*TET1*). Model: (Intercept), Subject groups (Control and DS), lymphocyte/monocyte.

*Impact of batch effects on total covariance*

Covariance parameters **Ln(*TET1*)**

|  |  | | |  |
| --- | --- | --- | --- | --- |
| Random effect | **Estimate** | **Std. Error** | **Z** | **p** |
|  |  |  |  |  |
| **Var(batch_TETs_TDG)** | 0.001 | 0.000 | 1.165 | 0.244 |

Covariance structure: variance components

**Supplementary Table 5.** Contribution of selected variables and covariates on *TET3* variation across control and DS subject groups †

*Test of Model Effects* **Ln(*TET3*)**

|  | **Type III** | | |
| --- | --- | --- | --- |
| Variables | **Wald**  **Chi-Square** | **df** | **p** |
|  |  |  |  |
| **(Intercept)** | 81.113 | 1 | < 0.001 |
| **Subject groups** | 3.451 | 1 | 0.063 |
| **Gender** | 0.123 | 1 | 0.726 |
| **Age** | 0.62 | 1 | 0.431 |

†Analysis was performed by GLM using linear model with identity link function considering as dependent variable: Ln(*TET3*). Model: (Intercept), Subject groups (Control and DS), gender, age (years).

**Supplementary Table 6.** Contribution of selected variables and covariates on *DNMT3A* variation across control and DS subject groups †

*Test of Model Effects* **Ln(*DNMT3A*)**

|  | **Type III** | | |
| --- | --- | --- | --- |
| Variables | **Wald**  **Chi-Square** | **df** | **p** |
|  |  |  |  |
| **(Intercept)** | 0.359 | 1 | 0.549 |
| **Subject groups** | 26.771 | 1 | < 0.001 |
| **Gender** | 3.633 | 1 | 0.057 |
| **Age** | 0.114 | 1 | 0.736 |
| **Ln(lympho/mono)** | 6.07 | 1 | 0.014 |

†Analysis was performed by GLM using linear model with identity link function considering as dependent variable: Ln(*DNMT3A*). Model: (Intercept), Subject groups (Control and DS), gender, age (years), Ln(lymphocyte/monocyte)

**Supplementary Table 7.** Contribution of selected variables and covariates on *TDG* variation across control and DS subject groups †

*Test of Model Effects* **Ln(*TDG*)**

|  | **Type III** | | |
| --- | --- | --- | --- |
| Variables | **Wald**  **Chi-Square** | **df** | **p** |
|  |  |  |  |
| **(Intercept)** | 64.53 | 1 | < 0.001 |
| **Subject groups** | 7.771 | 1 | 0.005 |
| **Gender** | 0.002 | 1 | 0.963 |
| **Age** | 0.005 | 1 | 0.945 |
| **Ln(lympho/mono)** | 2.434 | 1 | 0.119 |

†Analysis was performed by GLM using linear model with identity link‐function considering as dependent variable: Ln(*TDG*). Model: (Intercept), Subject groups (Control and DS), gender, age (years), Ln(lymphocyte/monocyte)

**Supplementary Table 8.** Contribution of selected variables and covariates on the difference in 5hmC levels across subject groups represented by DS, young and old people †

*Tests of Between-Subjects Effects*  **Ln(5hmC)**

|  | **Type III** | | |
| --- | --- | --- | --- |
| Source | **Sum of**  **Squares** | **df** | **p** |
|  |  |  |  |
| **Corrected Model**  **Intercept**  **Ln(lympho/mono)** | 0.872^a^  0.001  0.013 | 5  1  1 | 0.028  0.898  0.655 |
| **Country** | 0.131 | 1 | 0.163 |
| **Gender** | 0.012 | 1 | 0.667 |
| **Subject groups** | 0.792 | 2 | 0.003 |

^a^ R Squared = 0.101 (Adjusted R Squared=0.062)

†Analysis was performed by GLM using linear model considering as dependent variable: Ln(5hmC). Model: (Intercept), Subject groups (Young persons, Old persons, DS), gender, country of origin, Ln(lymphocyte/monocyte)

**Supplementary Table 9.** Contribution of selected variables and covariates on *TET1* variation across subject groups represented by DS, young and old people†

*Tests of Between-Subjects Effects* **Ln(*TET1*)**

|  | **Type III** | | |
| --- | --- | --- | --- |
| Source | **Sum of**  **Squares** | **df** | **p** |
|  |  |  |  |
| **Corrected Model**  **Intercept**  **Ln(lympho/mono)** | 4.834^a^  15.059  1.021 | 5  1  1 | 0.000  0.000  0.015 |
| **Country** | 0.305 | 1 | 0.178 |
| **Gender** | 0.008 | 1 | 0.825 |
| **Subject groups** | 3.372 | 2 | 0.000 |

^a^ R Squared = 0.169 (Adjusted R Squared=0.140)

†Analysis was performed by GLM using linear model considering as dependent variable: Ln(*TET1*). Model: (Intercept), Subject groups (Young persons, Old persons, DS), gender, country of origin, Ln(lymphocyte/monocyte)

**Supplementary Table 10.** Contribution of selected variables and covariates on *TET3* variation across subject groups represented by DS, young and old people†

*Tests of Between-Subjects Effects*  **Ln(*TET3*)**

|  | **Type III** | | |
| --- | --- | --- | --- |
| Source | **Sum of**  **Squares** | **df** | **p** |
|  |  |  |  |
| **Corrected Model**  **Intercept**  **Ln(lympho/mono)** | 2.008^a^  4.811  0.035 | 5  1  1 | 0.002  0.000  0.553 |
| **Country** | 0.003 | 1 | 0.860 |
| **Gender** | 0.029 | 1 | 0.589 |
| **Subject groups** | 1.786 | 2 | 0.000 |

^a^ R Squared = 0.125 (Adjusted R Squared=0.094)

†Analysis was performed by GLM using linear model considering as dependent variable: Ln(*TET3*). Model: (Intercept), Subject groups (Young persons, Old persons, DS), gender, country of origin, Ln(lymphocyte/monocyte)

**Supplementary Table 11.** Contribution of selected variables and covariates on *DNMT1* variation across subject groups represented by DS, young and old people†

*Tests of Between-Subjects Effects* **Ln(*DNMT1*)**

|  | **Type III** | | |
| --- | --- | --- | --- |
| Source | **Sum of**  **Squares** | **df** | **p** |
|  |  |  |  |
| **Corrected Model**  **Intercept**  **Ln(lympho/mono)** | 6.273^a^  11.153  0.068 | 5  1  1 | 0.000  0.000  0.536 |
| **Country** | 2.333 | 1 | 0.000 |
| **Gender** | 0.231 | 1 | 0.256 |
| **Subject groups** | 0.029 | 2 | 0.921 |

^a^ R Squared = 0.200 (Adjusted R Squared=0.172)

†Analysis was performed by GLM using linear model considering as dependent variable: Ln(*DNMT1*). Model: (Intercept), Subject groups (Young persons, Old persons, DS), gender, country of origin, Ln(lymphocyte/monocyte)

**Supplementary Table 12.** Contribution of selected variables and covariates on *TDG* variation across subject groups represented by DS, young and old people†

*Tests of Between-Subjects Effects*  **Ln(*TDG*)**

|  | **Type III** | | |
| --- | --- | --- | --- |
| Source | **Sum of**  **Squares** | **df** | **p** |
|  |  |  |  |
| **Corrected Model**  **Intercept**  **Ln(lympho/mono)** | 1.162^a^  9.691  0.012 | 5  1  1 | 0.066  0.000  0.739 |
| **Country** | 0.046 | 1 | 0.516 |
| **Gender** | 0.134 | 1 | 0.270 |
| **Subject groups** | 0.805 | 2 | 0.028 |

^a^ R Squared = 0.071 (Adjusted R Squared=0.037)

†Analysis was performed by GLM using linear model considering as dependent variable: Ln(*TDG*). Model: (Intercept), Subject groups (Young persons, Old persons, DS), gender, country of origin, Ln(lymphocyte/monocyte)

**Supplementary Table 13.** Contribution of selected variables and covariates on *TET2* variation across subject groups represented by DS, young and old people†

*Tests of Between-Subjects Effects*  **Ln(*TET2*)**

|  | **Type III** | | |
| --- | --- | --- | --- |
| Source | **Sum of**  **Squares** | **df** | **p** |
|  |  |  |  |
| **Corrected Model**  **Intercept**  **Ln(lympho/mono)** | 2.444^a^  12.466  0.768 | 5  1  1 | 0.765  0.000  0.370 |
| **Country** | 0.110 | 1 | 0.734 |
| **Gender** | 0.112 | 1 | 0.732 |
| **Subject groups** | 1.182 | 2 | 0.538 |

^a^ R Squared= 0.018 (Adjusted R Squared=-0.017)

†Analysis was performed by GLM using linear model considering as dependent variable: Ln(*TET2*). Model: (Intercept), Subject groups (Young persons, Old persons, DS), gender, country of origin, Ln(lymphocyte/monocyte)

**Supplementary Table 14.** Contribution of selected variables and covariates on *DNMT3B* variation across subject groups represented by DS, young and old people†

*Tests of Between-Subjects Effects* **Ln(*DNMT3B*)**

|  | **Type III** | | |
| --- | --- | --- | --- |
| Source | **Sum of**  **Squares** | **df** | **p** |
|  |  |  |  |
| **Corrected Model**  **Intercept**  **Ln(lympho/mono)** | 3.117^a^  53.042  0.032 | 5  1  1 | 0.284  0.000  0.801 |
| **Country** | 0.017 | 1 | 0.852 |
| **Gender** | 0.245 | 1 | 0.483 |
| **Subject groups** | 2.888 | 2 | 0.057 |

^a^ R Squared = 0.043 (Adjusted R Squared=0.009)

†Analysis was performed by GLM using linear model considering as dependent variable: Ln(*DNMT3B*). Model: (Intercept), Subject groups (Young persons, Old persons, DS), gender, country of origin, Ln(lymphocyte/monocyte)

**Supplementary Table 15.** Correlation between age and all analysed variables in young and old control subjects with/without the inclusion of DS samples†

|  | **Age years**  **(Y+O)** | **Age years**  **(Y+O+DS)** | **Age years**  **(Y+DS)** | **Age years**  **(O+DS)** |  |
| --- | --- | --- | --- | --- | --- |
| **Ln(5hmC)** | -0.2388  0.0329  * | -0.1264  0.1600  ns | -0.1629  0.1363  ns | -0.006704  0.9514  ns | Pearson r  p-value |
| **Ln(*TET1*)** | -0.3531  0.0003  *** | -0.136  0.0960  ns | -0.07667  0.4437  ns | 0.03461  0.7338  ns | Pearson r  p-value |
| **Ln(*TET2*)** | -0.09826  0.3283  ns | -0.05311  0.5172  ns | 0.02160  0.8294  ns | -0.02545  0.8026  ns | Pearson r  p-value |
| **Ln(*TET3*)** | -0.2458  0.0132  * | -0.06291  0.4428  ns | -0.1209  0.2262  ns | 0.1139  0.2618  ns | Pearson r  p-value |
| **Ln(*TDG*)** | -0.2669  0.0076  ** | -0.1357  0.0989  ns | -0.03307  0.7414  ns | -0.01763  0.8639  ns | Pearson r  p-value |
| **Ln(*DNMT1*)** | -0.2609  0.0088  ** | -0.292  0.0003  *** | -0.02406  0.8103  ns | -0.3703  0.0002  *** | Pearson r  p-value |
| **Ln(*DNMT3B*)** | -0.1758  0.0801  ns | -0.1136  0.1665  ns | 0.005367  0.9573  ns | -0.06287  0.5386  ns | Pearson r  p-value |

†Pearson’s correlation performed on different groups containing DS samples together with young (Y), 31-45 years, and/or old (O), 69-74 years, individuals. (*p < 0.05; **p < 0.05; ***p < 0.001).
